# The influence of Artificial Intelligence chatbots on consumer behaviour in digital marketing

**DOI:** 10.3389/frai.2026.1887433

**Published:** 2026-10-21

**Authors:** Roya Anvari, Saba Khergiani, Rokhsareh Mobarhan, Halimah Mohd Yusof

**Affiliations:** 1 The University of Georgia, School of Business and Administrative Studies, Tbilisi, Georgia; 2 Caucasus University, Caucasus School of Business, Tbilisi, Georgia; 3 Qazvin Islamic Azad University, Computer Science, Qazvin, Iran; 4 School of Human Resource Development, Universiti Teknologi Malaysia (UTM), Johor Bahru, Malaysia

**Keywords:** AI chatbots, anthropomorphic design, behavioural intention, interactivity, trust

## Abstract

**Introduction:**

Although artificial intelligence (AI)-powered chatbots are increasingly used in digital marketing, their adoption remains challenging because of limited understanding of the factors that shape consumer acceptance and behavioral intention. This study examines the roles of trust, chatbot interactivity, and anthropomorphic design in influencing consumers’ behavioral intention to use AI chatbot services, with a particular focus on consumers in Tbilisi, Georgia.

**Methods:**

A quantitative research design was employed using a purposive sampling approach to select consumers with previous experience using AI chatbot services. Data were collected through an online questionnaire from consumers who had previously used AI chatbot services provided by major organizations in Tbilisi, Georgia, between August and September 2025. A total of 275 valid responses were retained for analysis. The collected data were analyzed using Partial Least Squares Structural Equation Modeling (PLS-SEM) to assess the measurement model and test the hypothesized relationships among the study variables.

**Results:**

The findings show that chatbot interactivity and anthropomorphic design have significant positive effects on consumers’ behavioral intention. Trust significantly strengthens both relationships, indicating that higher levels of trust enhance the effects of interactivity and anthropomorphic design on behavioral intention.

**Discussion:**

The findings highlight the importance of interactive and human-like chatbot features and the moderating role of trust in encouraging consumers’ behavioral intention to use chatbot services in digital marketing.

## 1 Introduction

In our modern world, the importance of technological development has been clearly proved and marketing methodology has undergone serious streamlining. The discipline has broken its accustomed boundaries and is now communicating with digitally mediated platforms that enable direct interaction with discrete consumer cohorts. The emergence of the digital world has radically transformed communicative channels that businesses use to access their consumers and, as a result, made the digital marketing constructs a necessity in the present corporate environment. A digital marketing framework contains a range of strategies, such as social media marketing, email marketing, influencer marketing, content marketing, and search engine optimisation, whose interdependence and theoretical basis are thoroughly studied in recent academic literature (Akhlaq et al., 2025). Digital marketing has therefore become one of the central elements of strategic marketing portfolios in enterprises regardless of scale or industry.

Owing to the accelerated development of the digital platforms, the development of the aspects of technology and its ability to shape and influence the consumer’s behaviour, companies went over to the digital in the marketing aspects, and various digital channels serve to address the consumers. Digital marketing is a major driver of global business growth and consumer activities in the 21st century. The rapid growth of internet-related technologies, such as smartphones and social networking sites, has changed how businesses talk about their own worth, how they feel about customers, and how they track sales of goods and services (Fedotova, 2024).

The earlier works studied the effect of the Artificial Intelligence (AI) chatbots on consumer behaviour above all in the digital trade. Sheen (2020) opined that chatbots designed to mimic human conversation support customer service and personalise digital marketing experiences. Araujo (2024) stated that interactive systems strengthen trust and perceived usefulness, pushing the user to accept the AI technologies. In the same way, Nuss and Moon concluded that consumer trust in AI is promoted when chatbots demonstrate humanness through natural language understanding, empathy and contextual adaptation. Artasanchez and Joshi (2025) emphasised that an effective chatbot must be flexible, personal, available, and relatable to appropriately respond to the user and handle unforeseen interaction in a polite manner. Further, the literature also looked at the increasing role of conversational chatbots in the development of customer experiences and service delivery. Pillai and Sivathanu (2021) conceptualise chatbots as applications that simulate human conversation through the application of natural language processing, whereas Sheehan et al. (2020) view them as automated counsellors in decision-making. According to Melian-Gonzalez et al. (2021) and Mogaji et al. (2021), the use of chatbots in customer service will never be less than human interaction in the near future. Whereas Jang et al. (2021) and Agag (2025) draw focus to chatbots as self-service technologies that are facilitated by intelligent systems to increase efficiency in the sales, marketing, and support processes. Of more recent opinion, Agag et al. (2025) and other scholars thought that AI-driven chatbots are now vital in the tourism and hospitality sector, enhancing interaction with customers, saving money, and providing around-the-clock services.

However, there are still several lacunae in the field of consumer behaviour with respect to the impact of trust in AI robots on influencing consumers’ intention to use them. Most of the review focused on the aspect of the influence of anthropomorphic features on consumer behaviour and brand perception across various products. While the benefits of anthropomorphism are evident, further research is needed to understand how anthropomorphic design of AI chatbots influences consumers’ intentions and the joint impact of trust, interactivity, and anthropomorphic design on consumers’ behavioural intention to use AI chatbots in digital marketing. Recognising these gaps, there is a need for a more extensive study that incorporates these variables into a model that gives a better understanding of how the consumer develops trust and makes a decision to adopt the AI chatbot in the context of online shopping (Akhlaq and Ahmed, 2024).

In today’s digital world, AI chatbot technology has taken over and has made marketing easy. AI chatbots have become essential tools for assuring customers’ convenience and activities. However, customers face some difficulty in acceptance due to certain factors such as trust, interactivity and design perception. The degree to which they are satisfied with the acceptance of recent technology based on its implemented levels requires great attention. Although chatbots are invented to increase marketing efficiency, more than 70 % of the customers do not feel fully assured of their reliability and privacy, which makes their acceptance by the customers easy. Also, it should be further established how far the interactivity and human-like properties bestowed on AI chatbots are considerably affecting consumers’ attitudes towards them, but how far their effect on behavioural intention remains under study.

## 2 Theoretical background

### 2.1 Background literature

Digital marketing profoundly impacts consumer behaviour, which is influenced primarily by past experience with brands. The introduction of AI technology allows brands to interact with consumers in a different manner by means of data, automation and personalisation. One such feature of this technology is chatbots, which help engage in conversations between consumers and companies in a natural language. Research such as that of Gentsch (2023) concludes that firms invest in chat technology for enhancing conversations and also to be able to immediately provide required information. The findings by Xu et al. (2022) have illustrated that trust is essential for both low- and high-involvement purchasers interacting with AI-generated chatbots. The consumer perception of the structure of the communication, that chatbots are reliable or transparent, enables communication (Fox et al., 2020). Jain et al. (2023) further illustrate that when trust exists between the consumer and brand chatbots, there is a positive interaction which results in loyalty intentions and future interaction with the brand. For successful relationships with consumers, it is very important that firms identify which factors predispose consumers to trust firm AI chatbots.

#### 2.1.1 Trust in AI chatbot

Digital marketing has a significant impact on consumer behaviour, as consumers’ previous experiences with the brand are very influential. Machine learning technologies are extremely important as they bring data, automation, and customisation together to capture consumer attention. The use of chatbots is an extremely important aspect of technologies that enable brand-consumer communication in a more effective way, since they use natural language. In a study conducted by Gentsch (2023), it is shown that many brands make use of the AI chatbots in order to better the conversation rates and grant greater access to instant information. Xu et al. (2022) demonstrate that prominent characteristics influence the degree of trust the consumers have in connection with the dispensing of products and product information by means of AI chatbots. Ashfaq et al. (2020) also found that the use of the AI is greatly governed by opinions held by the consumers in connection with the chatbot characteristics, such as trustworthiness, sympathy and frankness, that are so dear to the consumer involved with communication with AI chatbots. Jain et al. (2023) showed that when consumers show trust in the chatbot of a brand, they have a satisfactory experience, resulting in loyalty to that particular service and a desire to communicate in the future with that chatbot. Thus, it is necessary for companies to recognise elements that have an important role in the building of consumer trust in AI chatbots in order to upgrade that area in their strategies of marketing communication. Table 1 presents a summary of previous tourism studies.

**Table 1:** Literature review: previous findings on Trust in AI Chatbot.

Source	Theoretical test	Methodology	Findings
Gentsch (2023)	Technology acceptance model	Conceptual and analytical review	Using AI chatbot integration for digital marketing communication was explored by Gentsch (2023). Discovered that AI chatbots can provide personalized and automated responses to customers. Chatbots are a more user-friendly and responsive way of engaging consumers.
Xu et al. (2023)	Unified theory of acceptance and use of technology	Quantitative analysis	Xu et al. (2023) examined how trust interfered with the quality of chatbot interaction and consumer dependability. Trust plays a significant role in consumers’ use of chatbots. Those who have greater trust are more likely to use chatbot services for purchasing.
Ashfaq et al. (2020)	Social presence theory	Mixed methods, interviews and experiments	Ashfaq et al. (2020) examined the psychological aspects of trust in AI-driven customer service systems. Chatbot trust was based on their ability to be reliable, compassionate, and transparent. Consumers’ confidence boosts their satisfaction and overall service experience.
Jain et al. (2023)	Relationship marketing theory	Quantitative methods	The impact of AI chatbot trust on brand loyalty was explored by Jain et al. (2023). Discovered that this trust led to emotional attachment and repeated visits with. Trusted chatbots have the potential to increase customer loyalty and long-term engagement.
Pillai and Sivathanu (2021)	Technology acceptance model	Descriptive analysis	Pillai & Sivathanu investigated the use of AI chatbots in online retail among consumers in 2021. Easy usage and perceived security were linked to increased consumer confidence and purchase decision making. Trust serves as a mediator between technological acceptance and behavioral intention.

#### 2.1.2 Anthropomorphic design

Artificial intelligence features that simulate human qualities are regarded as anthropomorphic design. This promotes good user experience in the case of artificial intelligence robots. This design helps foster warmer, more empathetic relationships between users and brands (Go and Sundar, 2019). The use of anthropomorphic robots is helpful in digital marketing to improve user experience and trust. The robots increase comfort and productivity by allowing interaction to be more in the form of human-style conversation with the use of social cues, such as humor and friendliness, which cause them to be perceived as more efficient and trustworthy (Ciechanowski et al., 2020). However, the other aspect of “The Uncanny Valley” (Luger and Sellen, 2016), indicates that if an engaging and conversational robot is too human-like, there would be discomfort experienced by the user or feelings of mistrust. The successful use of anthropomorphic robots would allow the brands to interact with the consumers through generating messages that are personalized and attach to the emotions of the consumer. This would result in higher levels of engagement and improved conversion rates (Zarouali et al., 2022). With the development of fast-paced digital landscape, the understanding of use and responses to communications with AI is vital. To this end, it is important to consider how consumers interpret, trust, and respond to AI-led communication in the rapidly changing digital market through anthropomorphic design. Table 2 presents a summary of previous studies.

**Table 2:** Literature review: previous findings on Anthropomorphic Design.

Source	Theoretical test	Methodology	Findings
Go and Sundar (2019)	The Computers Are Social Actors (CASA) Theory	Experimental study	The Computers Are Social Actors (CASA) Theory, which was formulated by Go and Sundar (2019), is the result of an experimental study that found chatbots with a human-like tone and emotional cues enhance user engagement and social presence
Ciechanowski et al.'s (2020)	Social presence theory	Quantitative approach	Ciechanowski et al.'s (2020) Social Presence Theory suggests that chatbots designed for humans are more likely to foster trust and satisfaction, particularly when users associate them with empathy and friendliness.
Luger and Sellen’s (2016)	Uncanny valley hypothesis	Conceptual study	According to Luger and Sellen’s (2016) conceptual study, the uncanny valley hypothesis suggests that chatbot designs with human-like features can result in discomfort and lower user confidence due to unrealistic expectations
Kim and Song’s (2023)	Human-computer interaction theory	Mixed-method approach	According to Kim and Song’s (2023) Mixed-method approach to Human-Computer Interaction Theory, the use of humor, emotion and facial avatars can enhance user experience by improving perceived usefulness. This paper presented an example of this concept in action.
Zarouali et al. (2022)	Parasocial interaction theory	Empirical research	Empirical research conducted by Zarouali et al. (2022) using the Parasocial Interaction Theory revealed that AI chatbots performing human-like communication strengthens consumer-brand relationships and increases buying intent, according to their study.

#### 2.1.3 Intention to use AI

Companies have many factors to consider about the perception of User Experience (UX) experienced by users from interaction with AI systems as well as the level of emotional connectedness that is developed by users toward AI systems in order to determine the best way for them to implement AI into their businesses from the interface of customer interaction via AI systems such as chatbots. The Technology Acceptance Model (TAM) states that a person perceives that the experience of using a particular AI technology will enhance the level of user productivity, convenience, and overall user experience as a result of the level of its use. Pillai and Sivathanu (2021) indicate that through the provision of instantaneous customer support, customised product offerings, and a human-like service experience, the use of AI chatbots is becoming more readily accepted in the digital marketing arena in that it provides an anticipated communication experience between human and machine. Researchers state that there are other factors that play a role in affecting the behavioural intention of humans toward AI systems, which include perceived enjoyment, security and emotional comfort (Mariani and Borghi, 2022). Further, based on that perception that the AI-based system is perceived to be reliable and knowledgeable and that it takes their preferences into account, consumers therefore exhibit a greater chance of addressing that AI-driven system (Gursoy et al., 2022). In conclusion, the motivation of consumers to use AI in the digital market is influenced by both the technological aspects of AI systems and the emotional and psychological experiences that result from the comforting experience. Table 3 presents a summary of previous studies.

**Table 3:** Literature review: previous findings on intention to use AI.

Source	Theoretical test	Methodology	Findings
Davis and Granić (2024)	Technology Acceptance Model (TAM)	Conceptual model	The TAM conceptual model, as described by Davis (1989) in a paper shortly thereafter, proposes that perceived usefulness and ease of use are the primary factors driving technology adoption and usage intention.
Dwivedi et al. (2021)	Unified Theory of Acceptance and Use of Technology (UTAUT)	Quantitative analysis	According to Dwivedi et al. (2021), the Unified Theory of Acceptance and Use of Technology (UTAUT) is quantitatively analyzed, and users’ level of readiness and belief in AI’s potential are crucial factors in motivating their interest in using it.
Pillai and Sivathanu’s (2021)	Trust theory	Qualitative analysis	According to Pillai and Sivathanu’s (2021) Trust Theory, an empirical study on online retailing, consumers are more likely to engage with AI chatbots for online communication due to their perceived value, reliability, and safety.
Mariani and Borghi (2022)	Resource based view	Case study	Mariani and Borghi (2022) revealed that organizations employing AI to enhance marketing experiences and increase consumers’ engagement with these technologies.
Gursoy et al. (2022)	Socio-technical systems theory	Mix method	Gursoy et al.’s Socio-Technical Systems Theory mixed-method research from 2022 revealed that users’ emotional comfort and confidence in AI responses lead to greater intentions for adoption and frequent use.

#### 2.1.4 Chatbot interactivity

Interactive chatbots refer to the continuous, back-and-forth flow of interaction of data between technology and human beings through the simulation of human interaction. Thus, it has the potential to create an atmosphere in which the consumer is more involved in the interface experience and is more satisfied because of contextualised communication, emotional support (Kvale et al., 2022) and/or feedback due to immediate responses to consumer input within the contextual boundaries of the interaction. The greater the human personality characteristics and responses to the consumer shown by the chatbot, the greater the perceptions of utility, credibility, and enjoyment of the consumer (Sheehan et al., 2020). The consumer can interact with interactive chatbots in order to receive personalised information from the brand and develop brand loyalty and brand engagement (Mariani and Borghi, 2022). Furthermore, interactive chatbots will result in a psychological sense of “social presence” by closing the gap created by human-to-human communication versus human-to-machine communication (Gnewuch and Reinkemeier, 2026), which results in a greater acceptance and increased use of these types of systems. Thus, the nature of the interactivity of chatbots should be investigated as revealing insight into consumer satisfaction and trust generation and consumer behavioural intentions in AI-mediated environments. Table 4 presents a summary of previous studies.

**Table 4:** Previous findings on Chatbot interactivity.

Source	Theoretical test	Methodology	Findings
Gnewuch and Reinkemeier (2026)	The social presence theory	Experimental study	The social presence theory, as researched by Gnewuch et al. (2023) through an experimental study, demonstrated that perceived social presence and user satisfaction are positively influenced by interactive features like responsiveness and empathy.
Sheehan et al.'s (2020)	Media richness theory	Survey-based study	Sheehan et al.'s (2020) Media Richness Theory Survey revealed that chatbots with better interactivity and conversational quality have a positive impact on users’ trust and engagement, according to their findings.
Kvale et al. (2022)	Human-Computer Interaction (HCI)	Qualitative analysis	Qualitative analysis of the Human-Computer Interaction (HCI) Framework by Kvale et al. (2022), demonstrated that both real-time interactivity and adaptive communication foster greater levels of user engagement/emotional connection.
Mariani and Borghi (2022)	The customer experience theory	Case study	The customer experience theory case study by Mariani & Borghi in 2022 demonstrated that the implementation of interactive chatbots can enhance customer loyalty by providing personalized and ongoing support.
Gursoy et al. (2022)	Technology Acceptance Model (TAM)	Mix method	A study conducted by Gursoy et al. in 2022 using the TAM mixed-method revealed that interactivity has a positive impact on both intent to use AI-driven chatbots and perceived usefulness.

### 2.2 Hypotheses development

#### 2.2.1 Anthropomorphic design and the intention to utilize AI

A design that is anthropomorphic is about how closely an artificial intelligence interface appears to be like a human; e.g., chatbots, or virtual assistants. A user will perceive an AI system that accomplishes tasks similarly to humans as being more relatable, trustworthy, and social than one which does not; this is based on Kim and Song (2023). The user’s perception of an AI system that performs tasks similarly to a human lead to users forming a greater emotional attachment to the AI; creates a smaller psychological distance between users and the AI; and fosters an intention by users to continue using the AI technology (Epley et al., 2007). Anthropomorphic Cues used in Digital Marketing also create positive User Attitudes; e.g., Customized Salutations; Empathy Expressed Through Language; Expressive Avatars (Moriuchi, 2021), etc., are all cues that create a sense of human warmth and trustworthiness in users. Additionally, when users interact with chatbots that have human-like social presence they experience higher levels of user satisfaction and adoption which makes users feel comfortable and confident in AI (Adam et al., 2021). Thus, Anthropomorphic Design has many implications regarding the Behavioral Intention to Continue Using AI-Based Systems. This supports the hypothesis: *H1 research indicates that Anthropomorphic Design is a favorable factor for Intention to Use AI*.

#### 2.2.2 Chatbot interactivity and intention to use AI

The interactivity of a chatbot represents its ability to facilitate an exchange that reacts to user interactions while providing quick answers, customised responses, and suggestions that pertain to the existing context of the user, thus generating an interaction type resembling conversation (Kvale et al., 2022). Additionally, the quality of user experience and engagement with AI technological systems will largely be impacted by the immediacy of response time fostered by AI systems, thereby increasing the perception of both usability and enjoyment (Gnewuch and Reinkemeier, 2026). Furthermore, it has been characterised that with the infusion of interactive functions into the chatbot, human users perceive the intelligence associated with the automated system as well as the social presence thereof (Sheehan et al., 2020). It has been mentioned that during higher interaction levels, higher satisfaction regarding the interaction will generate among users as well as greater willingness on behalf of users to utilise the AI systems and applications which they may interact with in the future (Mariani and Borghi, 2022). This supports the hypothesis: *A positive correlation exists between Chatbot Interactivity and the intention to use AI*.

#### 2.2.3 Trust in AI chatbot moderates a relationship between anthropomorphic design

The users’ perceptions of AI systems are directly linked to the extent of the trust the users hold in these systems in producing the desired result. But the users’ perceptions of the ability of AI systems to produce the desired results is very much determined by the trust that users place in the design elements of the systems (e.g., empathy, friendliness, human-type interaction) of the decisions to be made about making use of these systems. Thus, a recent study showed that users that had a high degree of trust in the pertinence of the anthropomorphic design of the systems to their acceptability of the systems would consider that digital interactions were less likely to be risky and hence less of a uncertainty than those who did not have a degree of trust in this field (Hajlici, 2022). Though a user lacks trust in the extent of how there is a use of anthropomorphic cues in AI this deficiency may lead to the users having the belief that these cues are manipulative or dishonest, they will tend to depart from using them as a result of deterrence in their intentions to behave there in response to the use of the technology. Similarly, AI systems would be able to possibly be fashioned to produce a positive emotional and cognitive response in the users marketed, tending to increase the users’ interest in expectation of the technology (Gefen et al., 2003). This supports the hypothesis: *The relationship between Anthropomorphic Design and the intention to use AI is moderated by Trust in AI Chatbot*.

#### 2.2.4 Trust in AI Chatbot moderates a relationship between anthropomorphic design and intention to use AI

Trust is important in modulating chatbot interactivity and user intent regarding AI. On one hand, interactivity has benefits in guiding users fully into engagement through communication in real-time and responsive manners but relies on the trust which users place in it regarding reliability and accuracy of information (Xu et al., 2023). Trust affects the enhanced interactivity in a positive direction, as it means that engagement from users is furthered through acceptance of suggestions by automated means and the ability to continue the closeness of relational interactions with the AI in question (Mariani and Borghi, 2022). However, the reverse can be said for low levels of trust, which deter the perceived advantages of interactivity. The final factor inherited in this is that the chatbot will have responsive aspects, but the issues of reliability must be taken into account. In other words, the relationship between interactivity and trust affects the overall behavioural intent to continue to interact with AI chat services. This supports the hypothesis: *Trust in AI Chatbot moderates the relationship between chatbot interactivity and intent-to-use using AI*. Based on the above discussion, the following conceptual framework was developed (Figure 1).

**Figure 1:**
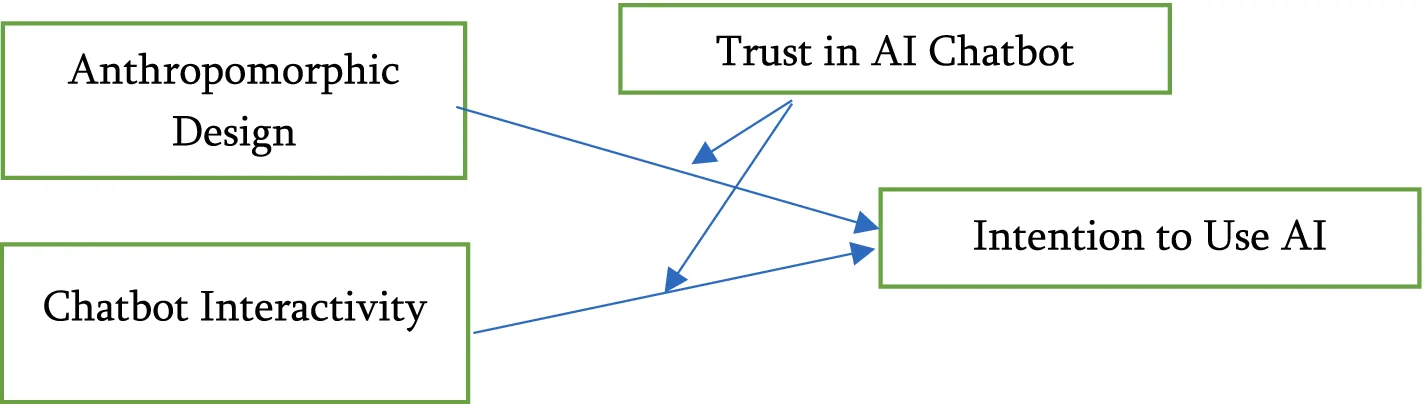
Conceptual framework.

## 3 Methods

The section will provide a description of the research design, data collection process, population, sampling method, research variables and the methods to be employed in the study of the population. To examine the issue of how modern consumer behaviour in Tbilisi, Georgia, is influenced by digital marketing approaches, especially the adoption of AI-based chatbots, the research uses quantitative research. With the help of the cross-sectional survey design, the retrospective data were gathered to examine the consumer attitudes to trust, interactivity, anthropomorphic design and intention to use AI chatbots. This research design is appropriate since it enables the researcher to gather primary data on consumers that utilise chatbot technologies in other industries, such as banking, retailing, e-commerce, telecommunications, and electronics.

### 3.1 Research design

The study is founded on the descriptive survey design. The right conception of the study is explained by the attention to the analysis of the influence of AI-supplied chatbots, in particular digital marketing strategies, on the current consumer practices in Tbilisi, Georgia. The study will utilise a survey study as its research method to gather primary data of organisational chatbots with consumers who use them in different sectors, which include banking, retail, e-commerce, telecommunications and electronics. Through such a design, it is possible to have quantitative data on user experiences, satisfaction levels, and behavioural directives of AI-managed digital marketing campaigns.

### 3.2 Data collection procedure

The collection of data was carried out through a well-structured online questionnaire administered by Google Forms, while maintaining efficiency, accessibility, and affordability. Two parts made up the instrument: Section A (Demographic Information): Gathers respondents’ backstory, such as their age, gender and educational attainment or occupation [Letter]. In Section B (Study Variables), the consumer is evaluated on their perception of AI-powered chatbots, including four dimensions: trust, interactivity, anthropomorphic design, and intention to use. The questionnaire was given out from August to September 2025 through Google Forms.

They rated the items out of five according to Likert scale, with 1 expressing Strongly Disagree and 5 Strongly Agree. This was a quantitative measure which enabled more subtle evaluation of respondent attitudes. Surveys were mainly done through the electronic mail. The use of complementary engagement channels such as WhatsApp, use of different social media platforms, and personalized newsletter were utilized to get maximum reach and involvement among target demographic.

### 3.3 Population of the study

The current study was designed to be inclusive of consumers who have interacted with AI-based chatbots in four specially chosen institutions located in Tbilisi, Georgia. These institutions were chosen on the basis of their superior integration of chatbot technology in the customer interaction and service delivery areas. According to an estimate from TBC Bank (Banking Sector, Table 5), there were 100,000 active users of digital apps and financial services chatbots. According to Elit Electronics (Retail electronics company), there were an estimated 25,000 users of chatbots who could assist with product inquiries and online purchase orders. 40,000 potential customers were estimated to be using customer service chatbots for promotions and delivery at the Magniti Supermarket Chain (Retail/Grocery). According to Silknet (Telecommunications), the usage of chatbots for data plans, billing, and technical assistance was estimated at 70,000 users per day for Geocell.

**Table 5:** Population.

Organization	Sector	Estimated AI-powered chatbot users
TBC bank	Banking/Financial	100,000
Elit electronics	Electronics/Retail	25,000
Magniti supermarket chain	Retail/Grocery	40,000
Geocell by silknet	(Telecommunications)	70,000
Total		235,000

Sampling technique and sample size.

The study used a purposive sampling approach, specifically choosing the sample that was purposely chosen, consisting of participants who had previous direct interactions with AI-driven chatbot systems that are functioning in the target corporate organisations.

*n* = *N*/(1 + *Ne*^2^) Where:

*n* = Signifies sample size.

*N* = Signifies population size.

*e* = Signifies margin of error (it could be 0.10, 0.05 or 0.01).

*n* = 235,000/[1 + 235,000 (0.05)^2^].

*n* = 235,000/[1 + 235,000 (0.0025)].

*n* = 235,000/(1 + 587.5).

*n* = 235,000/589.

*n* = 399.

Therefore, 339 respondents form the sample size for the study. But for purpose of the study 307 respondent was randomly selected from the sample population to form the sample population, questionnaire was distributed to 307 participated to collect data for the study.

### 3.4 Variables and measures

The constructs of the study were applied by utilizing validated and modified measures from past studies: 1.

#### 3.4.1 Trust in AI Chatbots

This construction was measured according to the work of Glikson and Woolley (2020). It measures users’ knowledge, trustworthiness and confidence in the integrity and dependability of AI chatbot systems. This measures the degree to which chatbots are deemed reliable, honest, and advantageous to users. Users’ confidence and perceived security when engaging with AI systems were assessed using six items to measure their trust in AI chatbots. In fact, as an example, the argument that AI chatbots demonstrate a high level of reliability is debated at the scholarly level.

#### 3.4.2 Chatbot interactivity

This construct was measured using modified items in Gomes et al. (2025). The chatbots’ interactivity summarizes their ability to interact with users by responding and communicating with them in a dynamic and personalized manner. This feature demonstrates the attentional acuity of the chatbot and its ability to provide relevant feedback and personalized experiences during service provision, simulating human-customer interactions. Chatbot interactivity was measured using three dimensions, namely responsiveness and user engagement. The findings were later put up. One of the chatbot service agent attributes allows the customers to be attended to on a one-on-one basis.

#### 3.4.3 Anthropomorphic design

The construct was operationalized and measured according to the methodology that was outlined by Polyportis and Pahos (2024). It measures how human-like chatbots are—in terms of natural language style, situational awareness, expressiveness, and visual/verbal/conversational finesse. The anthropomorphic design suggests that the users can connect with the chatbot as understanding, friendly, and human in communicative style. Five items were used to assess the ability of the chatbot to imitate human behaviour in the course of interaction. One such product outcome is that the chatbot looks more humane than human.

#### 3.4.4 Intention to use AI

The evaluation of this construct was done through the data presented by Chin et al. (2022). The research questions potential user behaviour and their intentions to use AI chatbot services in different situations. Results outline the satisfaction, perceived usefulness, and intention to experiment with AI technologies levels. The tendency to utilise AI was tested with three levels of continuum and with a special focus on the probability of future use, as well as long-term involvement. As an example, the constructs were measured using a 5-point Likert scale that was created to measure the intentions of using AI chatbot services in the future. To confirm the validity and reliability of the survey instrument, a strict psychometric analysis, including the measures of internal consistency (Cronbach’s alpha >0.70), composite reliability (CR > 0.70), and an average variance extracted greater than 0.50, will be conducted to prove the convergent validity. Discriminant validity will be evaluated with the use of Fornell-Larcker criteria.

#### 3.4.5 Method of data analysis

Descriptive and inferential statistics will be employed to analyze the data obtained from structured questionnaires. A summary of demographic information and general perceptions about AI-powered chatbot interactions will be provided using descriptive statistics like mean, frequency, percentage, and standard deviation. The use of Chi-square tests and regression analysis will be employed to conduct Inferential statistics, while statistical analysis using Statistical Package for the Social Sciences version 26.0.

## 4 Results

### 4.1 Descriptive statistics

A demographic overview of the respondents is as follows: the largest number of respondents are in the 25–34 age group (52.4%) and predominantly male (54.4%), with a large number of the sample being self-employed (56.7%) and having completed a bachelor’s degree (62.3%). The majority of the sample have already utilised an AI chatbot (86.3%), and the primary uses have been for university or online educational purposes (31.6%), customer service (28.7%), and to assist with their online shopping (27%). This sample reflects a young, educated population which utilises technology at a high level (Table 6).

**Table 6:** Demographic variables.

Variable		Freq	Percent%
Age	18–24	76	24.8
	25–34	161	52.4
	35–44	20	6.5
	45+	50	16.3
	Total	307	100.0
Gender	Male	167	54.4
	Female	140	45.8
	Total	307	100.0
Employment status	Student	68	22.1
	Employed	43	14.0
	Self employed	174	56.7
	Unemployed	22	7.2
	Total	307	100.0
Education level	High school	27	8.9
	Bachelor’s degree	190	62.3
	Master’s degree	84	27.5
	Doctorate or higher	6	1.9
	Total	307	100.0
Have you ever used an AI chatbot?	Yes	265	86.3
	No	42	13.7
	Total	307	100.0
In which field have you mostly used AI chatbots?	Banking and financial services	39	12.7
	University and online education	97	31.6
	Online shopping	83	27.0
	Customer support services	88	28.7
	Total	307	100.0
Field work			

Table 7 presents the results of construct reliability and convergent validity for Anthropomorphic Design (ANTH), Behavioural Intention (BINT), Interactivity (INTER), and Trust (TRUST). The composite reliability values (rho_c) for BINT (0.891), INTER (0.852), and TRUST (0.839) all exceed the recommended threshold of 0.70, indicating good internal consistency and reliability of these constructs. The acceptable level of composite reliability in ANTH is reflected in a value of 0.741. This suggests that the indicators used to measure this construct are sufficient to measure it even though its value for Cronbach’s Alpha was low. Cronbach’s Alpha indicates high levels of internal consistency for the measures of BINT (0.815), INTER (0.739), and TRUST (0.769). The Cronbach’s Alpha for ANTH (0.588) is lower than the recommended 0.70 threshold, which indicates weak internal consistency between the indicators measuring this construct. Regarding convergent validity, the AVE values for BINT (0.731) and INTER (0.658) both exceeded the 0.50 minimum cutoff and therefore provide evidence that these two constructs have adequate convergent validity. ANTH (0.43) and TRUST (0.475) had AVE values less than 0.50, which indicates that at least some of the indicators measuring these constructs did not well enough capture the variance of their respective latent variables. This relatively weak AVE may influence the strength of their relationships within the structural model.

**Table 7:** Construct reliability and validity.

Variable	Cronbach’s alpha	Composite reliability (rho_a)	Composite reliability (rho_c)	Average variance extracted (AVE)
ANTH	0.588	0.750	0.741	0.433
BINT	0.815	0.817	0.891	0.731
INTER	0.739	0.755	0.852	0.658
TRUST	0.769	0.804	0.839	0.475

The results of Table 8 are used to test the discriminant validity with the use of the Fornell-Larcker Criterion. Each construct’s square root of AVE values is higher than the correlation values for the respective constructs. The diagonal values in Table 3 represent the constructs’ square root of AVE. ANTH (0.658), BINT (0.855), INTER (0.811), and TRUST (0.689) are all greater than their correlations with one another. The results from the Fornell-Larcker criterion show that the constructs were clearly distinguished by respondents as such. Although AVE values were relatively low for ANTH and TRUST, the Fornell-Larcker criterion shows that discriminant validity was attained.

**Table 8:** Discriminant validity Fornell-Larcker criterion.

Variable	ANTH	BINT	INTER	TRUST
ANTH	0.658			
BINT	0.592	0.855		
INTER	0.616	0.522	0.811	
TRUST	0.591	0.483	0.513	0.689

The data in Table 9 shows discriminant validity for all of the total constructs in this study. This is shown by the fact that the diagonal elements of each construct were larger than the correlations between each construct and its peers. These moderate levels of correlation exist among anthropomorphic design, behavioural intention, interactivity, and trust; for example, ANTH–INTER = 0.853 and ANTH–TRUST = 0.807, which would suggest that these variables do not represent overlapping concepts. Therefore, it has been demonstrated that each of the constructs measured different concepts and, therefore, provides support for the measurement model’s appropriateness for structural equation modelling.

**Table 9:** Discriminant validity of overall constructs.

Variable	ANTH	BINT	INTER	TRUST
ANTH				
BINT	0.751			
INTER	0.853	0.669		
TRUST	0.807	0.583	0.669	

The outer loadings of the indicators representing Anthropomorphic Design (ANTH), Behavioural Intention (BINT), Interactivity (INTER), and Trust (TRUST) are presented in Table 10. The majority of the Anthropomorphic Design indicator loadings were sufficient, and they were greater than the 0.50 threshold that is suggested by the literature; those being ANTH1 (0.702), ANTH12 (0.818), ANTH3 (0.650), and ANTH5 (0.759), which indicate that the items associated with each indicator provide reliable representations of the construct. In contrast, ANTH4 has an extremely low and negative loading (−0.074), which indicates that it may be contributing little or no meaningful information to the representation of the construct and therefore potentially reducing the measurement reliability of this dimension. All behavioural intention indicators showed high loadings to the construct; BINT1 (0.841), BINT2 (0.896), and BINT3 (0.827) exceeded the threshold for acceptable loadings. The loadings of interactivity indicators ranged from 0.749 to 0.850 and thus indicated that the indicators provided robust representations of the construct. The loadings of the Trust indicators varied; while TRUST2 (0.774), TRUST3 (0.726), TRUST5 (0.789), and TRUST6 (0.801) had good loadings, TRUST1 (0.387) and TRUST4 (0.585) had poor loadings, which could account for the lower convergent validity reported for the Trust construct.

**Table 10:** Outer loading.

Variable	ANTH	BINT	INTER	TRUST
ANTH1	0.702			
ANTH12	0.818			
ANTH3	0.650			
ANTH4	−0.074			
ANTH5	0.759			
BINT1		0.841		
BINT2		0.896		
BINT3		0.827		
INTER1			0.832	
INTER2			0.850	
INTER3			0.749	
TRUST1				0.387
TRUST2				0.774
TRUST3				0.726
TRUST4				0.585
TRUST5				0.789
TRUST6				0.801

There was an overall moderate to strong positive correlation for all of the variables in the study. There were moderate positive correlations between ANTH and BINT (*r* = 0.585), as well as ANTH and INTER (*r* = 0.620), and ANTH and TRUST (*r* = 0.604). The same pattern was observed when examining relationships of BINT with INTER (*r* = 0.520) and TRUST (*r* = 0.470). As such, the findings suggest that as ANTH, INTER and TRUST increase so too does BINT for users of an AI chatbot service. Since all r values are less than threshold values for multicollinearity, the associations found in this study support the hypothesized relationships among these constructs (Table 11).

**Table 11:** Construct correlation matrix.

Variable	ANTH	BINT	INTER	TRUST
ANTH	1.000	0.585	0.620	0.604
BINT	0.585	1.000	0.520	0.470
INTER	0.620	0.520	1.000	0.513
TRUST	0.604	0.470	0.513	1.000

The results from Table 12 show the structural model results—the path coefficients, standard errors, means, *t*-values, and *p*-values for the proposed relationships. To begin with, the relationship between TRUST and BINT has a positive relationship as indicated by a *t*-statistic value of 2.513, a *p*-value of 0.012 and a path coefficient of 0.155; because the *p*-value (0.012) is less than 0.05, it can be concluded that a user’s trust in AI chatbots will have a positive effect on the user’s behavioural intent to use them. Thus, H1 is supported. Secondly, the INTER → BINT relationship is also positive, as illustrated by a *t*-value of 3.091, a *p*-value of 0.002 and a path coefficient of 0.216. Since the *p*-value is less than 0.01, it follows that interactivity of chatbots has a positive and significant effect on BINT. Thus, H2 is also supported. Thirdly, concerning H3 (ANTH → BINT), we find that there is a positive and very significant relationship between the anthropomorphic design of AI chatbots (ANTH) and BINT, as demonstrated by the path coefficient = 0.367, *t*-value = 5.645, and *p*-value = 0.000. These statistics confirm that the impact of ANTH on BINT is both large and significant at *p* < 0.01. Therefore, H3 is also supported. Fourthly, for H4 (TRUST, INTER, and ANTH → BINT), the total effect of trust, interactivity, and anthropomorphic design on behavioural intention was found to be significant, as shown by the path coefficient = 0.738; *t*-value = 5.762; *p*-value = 0.000. The combined effect of these factors is therefore significant and accounts for a substantial proportion of the variance in behavioural intention. Therefore, H4 is supported. Finally, regarding H5 (TRUST → ANTH → INTER), it is shown that there is a statistically significant indirect relationship between TRUST and INTER via ANTH due to the statistically significant effect of trust on ANTH and ANTH on INTER with a path coefficient of 0.065, a t value of 6.431, and *p* = 0.001. This is considered an indirect effect because trust has a positive effect on ANTH, and ANTH has a positive effect on INTER. Therefore, H5 is supported and demonstrates that trust affects consumers’ behaviour via their perception of design features that encourage interaction with AI chatbot services. Figures 2 and 3 show the PLS algorithm and bootstrapping results, respectively.

**Table 12:** Hypotheses testing.

Hypotheses	Path	Path coefficient	SD	Mean	*t*-value	*p*-value	Result
H1	TRUST→BINT	0.155	0.062	0.161	2.513	0.012	Accepted
H2	INTER→BINT	0.216	0.070	0.211	3.091	0.002	Accepted
H3	ANTH→BINT	0.367	0.065	0.372	5.645	0.000	Accepted
H4	TRUST_INTER_ANTH→BINT	0.738	0.197	0.744	5.762	0.000	Accepted
H5	TRUST→ANTH→INTER	0.065	0.070	3.091	6.431	0.001	

^***^p < 0.01, ^**^p < 0.05.

**Figure 2:**
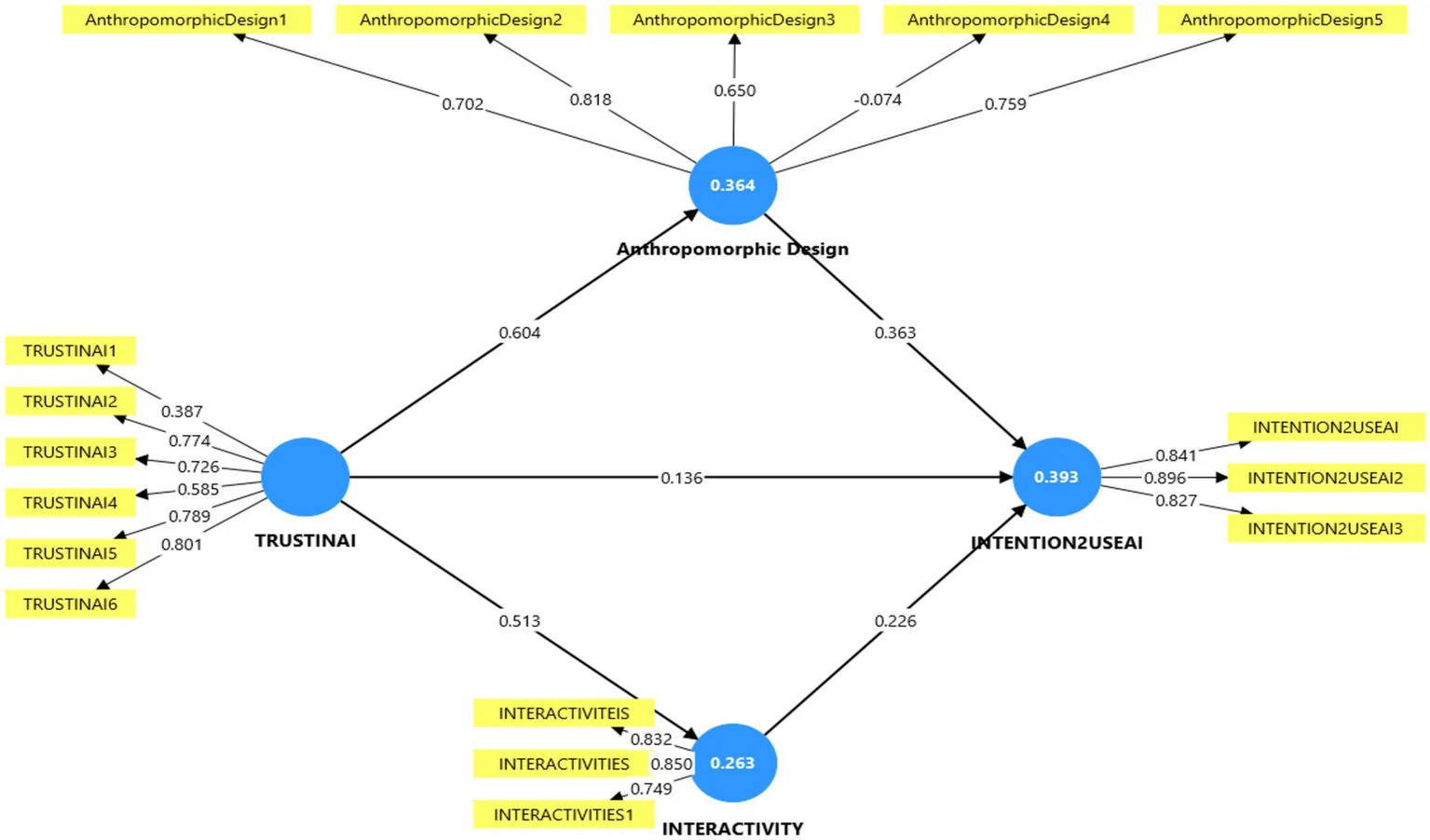
PLS algorithm.

**Figure 3:**
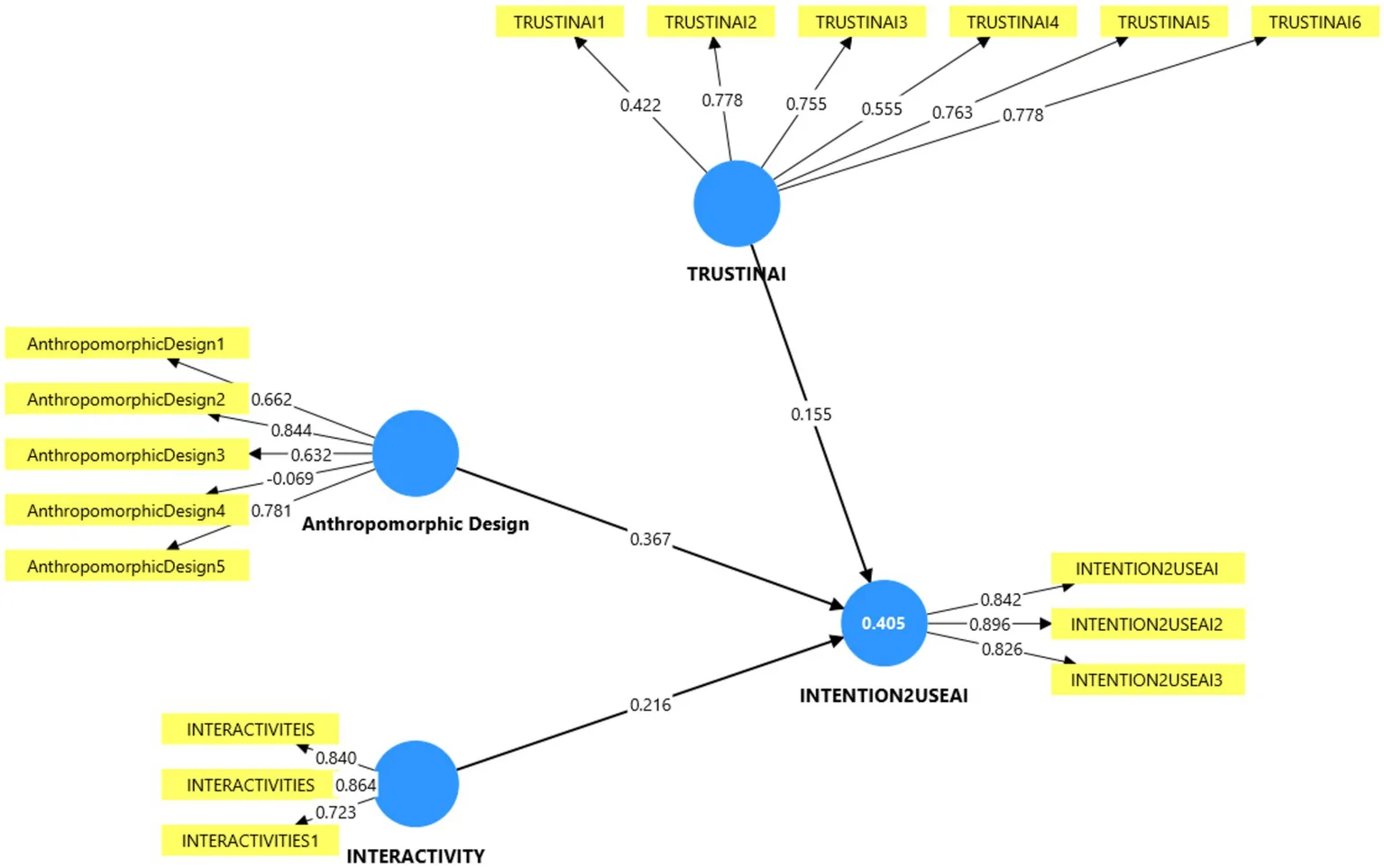
Bootstrapping results.

Results from Model Fit indicate that the PLS-SEM model fits well enough overall. The SRMR (saturated and estimated) of 0.077 is lower than the recommended maximum limit of 0.08. Moderate model fit is indicated by an NFI of 0.745, and d_ULS, d_G, and chi-square values are also indicative of a good model fit (Table 13).

**Table 13:** Model fit.

Fit index	Saturated model	Estimated model
SRMR	0.077	0.077
d_ULS	0.899	0.902
d_G	0.287	0.287
Chi-square	497.327	498.304
NFI	0.745	0.745

### 4.2 Discussion of findings

The empirical evidence shows that the trust in AI chatbots has a considerable positive impact on the behavioural intentions of consumers to embrace AI chatbot services in digital marketing. Such considerations imply that AI chatbots can be made more credible and reliable when appropriately designed to advance the best interests of users, which will make them more acceptable. These findings can be related to similar studies carried out in the past that have highlighted the critical role of trust as a fundamental factor that dictates the section of consumers to adopt technology. The original contribution of Gefen et al., 2003 and recent articles (2021) complain that trust reduces the perceived risk and uncertainty in the online interaction and makes wider adoption possible. Similarly, McKnight and associates also proved that trust is a predictor of user participation in online platforms, which supports the idea that, in digital marketing, trust is still a core part of consumer-AI relationships. The interactivity of chatbots comes out as a prominent element of influencing the behavioural choices that consumers make in the use of AI chatbot services, as shown by hypothesis H2. Quick-replied chatbots with two-way communications and resembling the natural human dialogue are hypothesised to increase user engagement and adoption intentions. In the way modern chatbot prototypes are designed, this premise is expressed. The results are consistent with the literature of Sundar, Bellur, Oh, Jia, and Kim, as they hypothesised that attributes of the interactive system increase the level of involvement and control among users, which contributes to positive behavioural results. Also, Ashfaq et al. (2020) discovered that the more interactive the chatbot is, the more satisfied the user will be and will be willing to use AI-enhanced services again. In general, the current research supports the idea that interactivity is a vital factor in determining the effect of AI chatbots on consumer behaviour within the context of digital marketing. In addition, these results demonstrate that an anthropomorphic approach to designing AI chatbots has a very strong, statistically significant positive impact upon consumer behavioral intention to use AI chatbot services. As such, it can be inferred that chatbots that are designed with human-like characteristics (e.g., conversational tone, emotion, name or avatar) will be more readily accepted than those without. Thus, this finding supports the CASA (Computers Are Social Actors) paradigm proposed by Reeves and Nass (2006) that individuals treat technological entities exhibiting human-like attributes as they would other humans. In similar fashion, Epley et al. and Kim and Sundar (2012) demonstrated through their respective studies that the use of anthropomorphism can enhance the perceived social presence, warmth and engagement users experience from utilizing a service. The strong effect observed in this study highlights the importance of human-like design elements in shaping positive consumer responses to AI chatbots. As the findings indicate from Hypothesis Four, there is an impact of trust, interactivity, and anthropomorphic design as a collective on consumers’ behavioural intention to use AI chatbot service in digital marketing. In other words, it seems that the most effective way to understand consumers’ willingness to engage in adopting new technology like AI chatbot services is by considering all three of these variables, and not separately. The findings are consistent with the TAM (Technology Acceptance Model) and extensions to the TAM, which suggest that numerous cognitive and affective factors contribute to technology usage behaviour (Davis, 1989; Venkatesh et al., 2003). Therefore, this study has provided a broader perspective of how AI chatbots can influence consumer intentions through the combined effects of trust, system interactivity, and anthropomorphic cues. In addition, this combined effect supports the necessity for digital marketers to take a systematic design and trust-building strategy to successfully deploy AI chatbot technologies.

Moreover, H5 reveals that trust significantly moderates the relationship between anthropomorphic design and chatbot interactivity, indirectly influencing consumers’ behavioural intention. The path coefficient (0.065, *t* = 6.431, *p* = 0.001) suggests that consumers’ perceptions of a higher level of trust will enhance the positive effect of anthropomorphic design on interactivity. In this regard, since consumers who have higher levels of trust in a system will be more likely to perceive human-like chatbots as being responsive, the results suggest that trust in the system will result in greater consumer engagement with human-like chatbots. The findings also support previous research that found that trust increases users’ cognitive and affective evaluation of technology (Gefen et al., 2003).

## 5 Theoretical implications

The research contributes to developing the theory regarding the AI adoption process, digital marketing behaviours, and the behaviour in interactions with both humans and machines. The study builds upon the TAM model by determining that trust and anthropomorphic design are important external variables which can positively affect behavioral intentions. Current literature has expanded upon the original TAM construct of perceived usefulness and perceived ease of use to be applicable to AI-based systems; therefore, this study is also placing a high level of emphasis on trust as an underlying psychological factor to drive acceptance.

The study’s results add to HCI theories by showing that human computer interface components such as human-like language, emotional cues, and natural communication interfaces improve user acceptance. This supports Nass and Moon’s Computers Are Social Actors (CASA) theory, which posits that humans instinctively respond socially to machines exhibiting human-like traits. This study demonstrates the role of anthropomorphism as a critical determinant of users’ intentions toward AI-based applications in digital marketing. In addition to contributing to the Unified Theory of Acceptance and Use of Technology (UTAUT), the research also demonstrated that user expectations for interaction with technology are influenced equally by both “human” (e.g., human-likeness) and “performance” (e.g., trust) factors when using technology; therefore, based on the results of this study (i.e., very little interaction), researchers will need to revise many existing theoretical models to better account for how people expect to interact in environments where they are likely to be interacting with automated or artificial intelligence (AI)-based systems. The study brings together results from TAM, HCI and UTAUT to provide a broader understanding of how users interact with AI-based chatbots.

## 6 Managerial and practical implications

The research presents critical practical insight into business, digital marketing and artificial intelligence (AI) development. First, because trust in chatbots is a significant determinant of intended use of chatbots; therefore, organisations need to focus on developing transparent, secure and reliable AI systems by making clear communication of their privacy policy, improving the accuracy of the system, limiting the number of errors made by the system, and making sure the chatbot acts ethically and in a consistent manner.

Even the empirical evidence shows that the sheer existence of chatbot interactivity does not have a statistically significant effect on the behavioural intentions of users. Therefore, managers ought not to waste resources on the extravagant interactive processes that do not directly provide a benefit to the consumer experience. Rather, they should focus on key features that maintain business effectiveness in terms of clarity and reliability. A designed chatbot with an easy, quick, and accurate format is likely to be more beneficial to the organisation than the one that involves a lot of interaction.

The research under consideration suggests that anthropomorphic design has a significant influence on intention, which means that the acceptance of users can be increased as a result of a wise consideration of the properties of a human. To produce chatbots that appeal to the user, companies should incorporate functionalities like natural language processing tools, friendliness, emotional intelligence, and customised messaging. The inclusion of these aspects does not only help in alleviating the fears of artificial intelligence but also makes online communication more active.

With the decisive role of trust, interactivity, and anthropomorphism in the general development of the chatbots, there is an urgent need to enable a holistic approach to enterprises. Using one determinant would not be effective enough to change the behaviour of consumers. The general aim of any digital marketing business must then be to create AI systems that combine reliability, usability, and human interaction indicators, therefore successfully steering consumer decision-making.

The continuous feedback stream of users, the thorough method of optimization of chatbot activity, and the improvement of the design elements that meet consumer demands require the close cooperation of the AI developers and marketing professionals. This approach will help promote the preservation of customer satisfaction and stimulate the acceptance of chatbot-based services in the long term.

## 7 Limitations and further research directions

Although the study has some merits, it is not without severe limitations. Primarily, the lack of statistically significant results with regard to chatbot interactivity may be attributed to methodological limitations such as the inadequacy of survey items or the incomplete understanding of the construct of interactivity by respondents. For that reason, more studies are needed to refine and increase the number of these measurement instruments to improve their reliability.

Future studies should combine behavioural measures based on actual chatbot interaction to produce more objective measures. Furthermore, the sample of the study was limited to a certain group of people, which may hinder the generalisation of the study results. The replication of the investigation in diverse geographical locations, industries, and demographic strata would improve its generalisability. Furthermore, the use of a cross-sectional questionnaire limits the capacity to track changes in the beliefs or behaviours of respondents over time; a longitudinal research design would produce a more accurate picture of the evolution of attitudes towards AI chatbots over time.

The use of self-reported information can create problems of potential bias, such as from social desirability and subjective interpretation. In addition, prospective research should investigate possible moderating factors—digital literacy, privacy fears, culture, personality factors, or prior exposure to artificial intelligence. The constructs like trust, anthropomorphism, and interactivity are expected to impact the intentions of the users to use chatbots. Qualitative inquiry will enable the researcher to discover even more psychological factors that drive the uptake of AI and, hence, provide a more beautiful picture of consumer behaviour.

## 8 Conclusion

The current research questioned whether trust, interactivity, and human-like design in AI chatbot services affect how likely consumers are to use digital marketing programs. Results show that trust and human-like design are key factors in influencing behaviour, while interactivity does Results indicate that trust and anthropomorphic design are important predictors of behavioural intention, and interactivity does not make any statistical differences in terms of utilisation. The joint effect analysis also underlines the importance of incorporating several attributes in order to maximise the effectiveness of chatbots. The investigation sheds light on the theoretical underpinning of the adoption of AI through the synthesis of the insights of the technology acceptance, human-computer interaction, and digital marketing literature. The empirical knowledge as such provides practical advice to those practitioners who may want to build chatbots that can enhance trust, user experience, and expand acceptance.

Though with a few methodological weaknesses, the study serves as a good guiding factor for future research and can help organisations to leverage AI chatbots in digital marketing endeavours. Using AI systems that are reliable and easy for machines to understand would help companies enhance the online experience for users by implementing trustworthy, human-like, and well-designed systems.

## Data Availability

The original contributions presented in the study are included in the article/supplementary material, further inquiries can be directed to the corresponding author.
